# A Novel Stress-Induced Sugarcane Gene Confers Tolerance to Drought, Salt and Oxidative Stress in Transgenic Tobacco Plants

**DOI:** 10.1371/journal.pone.0044697

**Published:** 2012-09-11

**Authors:** Kevin Begcy, Eduardo D. Mariano, Agustina Gentile, Carolina G. Lembke, Sonia Marli Zingaretti, Glaucia M. Souza, Marcelo Menossi

**Affiliations:** 1 Laboratório de Genoma Funcional, Departamento de Genética, Evolução e Bioagentes, Instituto de Biologia, Universidade Estadual de Campinas, Campinas, Brazil; 2 Laboratório de Transdução de Sinal, Departamento de Bioquímica, Instituto de Química, Universidade de São Paulo, São Paulo, Brazil; 3 Unidade de Biotecnologia, Universidade de Ribeirão Preto, Ribeirão Preto, São Paulo, Brazil; University College Dublin, Ireland

## Abstract

**Background:**

Drought is a major abiotic stress that affects crop productivity worldwide. Sugarcane can withstand periods of water scarcity during the final stage of culm maturation, during which sucrose accumulation occurs. Meanwhile, prolonged periods of drought can cause severe plant losses.

**Methodology/Principal Findings:**

In a previous study, we evaluated the transcriptome of drought-stressed plants to better understand sugarcane responses to drought. Among the up-regulated genes was *Scdr1* (sugarcane drought-responsive 1). The aim of the research reported here was to characterize this gene. *Scdr1* encodes a putative protein containing 248 amino acids with a large number of proline (19%) and cysteine (13%) residues. Phylogenetic analysis showed that ScDR1is in a clade with homologs from other monocotyledonous plants, separate from those of dicotyledonous plants. The expression of *Scdr1* in different varieties of sugarcane plants has not shown a clear association with drought tolerance.

**Conclusions/Significance:**

The overexpression of *Scdr1* in transgenic tobacco plants increased their tolerance to drought, salinity and oxidative stress, as demonstrated by increased photosynthesis, water content, biomass, germination rate, chlorophyll content and reduced accumulation of ROS. Physiological parameters, such as transpiration rate (E), net photosynthesis (A), stomatal conductance (gs) and internal leaf CO_2_ concentration, were less affected by abiotic stresses in transgenic *Scdr1* plants compared with wild-type plants. Overall, our results indicated that *Scdr1* conferred tolerance to multiple abiotic stresses, highlighting the potential of this gene for biotechnological applications.

## Introduction

Crop yield is negatively influenced by a large number of environmental factors. Abiotic stresses are a primary cause of reduced crop growth and productivity, and of these, drought, salinity, temperature, aluminum toxicity, flooding, pollution and radiation are among the most frequent [1]. It is estimated that stresses may reduce productivity by up to 70% [2], [3]. Abiotic stress affects the plant at different levels [4] by reducing CO_2_ assimilation rates, leaf cell size, rate of transpiration, water potential, plant growth rate and stomatal opening [5], which affect photosynthesis both directly and indirectly by inducing physiological changes that can lead to plant death [6].

There is a constant demand, especially in developing countries, for increased crop production to serve the increasing needs of the population. These needs can be satisfied by increasing the cultivated area (i.e., planting in regions that were not previously used) or by increasing crop productivity. To guarantee a sustainable crop yield, it is necessary to design and develop better crop varieties that can tolerate the harmful effects of constantly changing environmental factors. Thus, it is essential to identify novel and functional candidate genes that may lead to stress tolerance and improved productivity.

Sugarcane is an important tropical and subtropical crop that is used primarily to produce ethanol and sugar; however, there is also important economic activity associated with the production of other products, including rum, animal feed and molasses [7], [8]. In large areas of sugarcane-growing regions, irrigation cannot satisfy plant water requirements during cane formation, which results in low yields [9]. Our understanding of plant responses to stresses has improved significantly due to advances in the related areas of genetics, physiology and molecular biology [10], with increasing evidence that some genes have the potential to reduce the effects of resource limitation imposed on crops [11]. Several studies aiming to understand plant responses to water deficit have been conducted (for reviews see [4], [12], [13]). The currently available data indicate that plant responses to abiotic stress are complex, involving many different genes that produce responses at the biochemical, physiological and molecular levels [4], [14]. These genes are classified into four main categories: genes involved in signaling cascades and transcriptional control, genes that function directly in the protection of membranes and proteins, genes involved in water and ion uptake and transport, and genes of unknown function [15]. The identification and characterization of genes associated with plant responses to stress are crucial to the development of new cultivars with improved tolerance. Towards this end, several stress-induced genes have been overexpressed in transgenic plants, including a gene encoding a hybrid-proline-rich protein from the pigeon pea that confers tolerance to multiple abiotic stress in *Arabidopsis*; a DREB/CBF factor from wheat, which enhances tolerance to drought and cold stress in barley and wheat; *TSRF1*, which is an ERF transcription factor from tomato that improves tolerance to drought in rice and a novel sugarcane ethylene responsive factor (ERF), which enhances salt and drought tolerance in tobacco plants [16], [17], [18], [19].

In sugarcane, the expression profiles of 1,545 genes in plants exposed to drought, phosphate starvation, herbivory, methyl jasmonate, abscisic acid and two N_2_-fixing endophytic bacteria were evaluated by Rocha et al. [20]. More recently, approximately 1,670 genes were found to be differentially expressed in sugarcane plants exposed to water deficit [21]. A wide array of metabolic pathways was reported to be affected by these treatments, based on the numerous genes that were modulated in response [20]. Interestingly, drought was the treatment that caused the most changes in the sugarcane transcriptome. The cDNA array used in these experiments also included several genes of unknown function, some of which were detected as differentially expressed upon drought stress. Here, we have sought to characterize one of these genes. This gene was named *Scdr1*, for sugarcane drought-related 1. Phylogenetic analysis indicated that this gene was present prior to the divergence between dicots and monocots. The expression pattern of *Scdr1* in four sugarcane cultivars showed that it is regulated by drought. The overexpression of *Scdr1* in transgenic tobacco plants induced enhanced tolerance to drought, salt and oxidative stress.

## Results

### *Scdr1* Expression in Response to Drought

**Figure 1:**
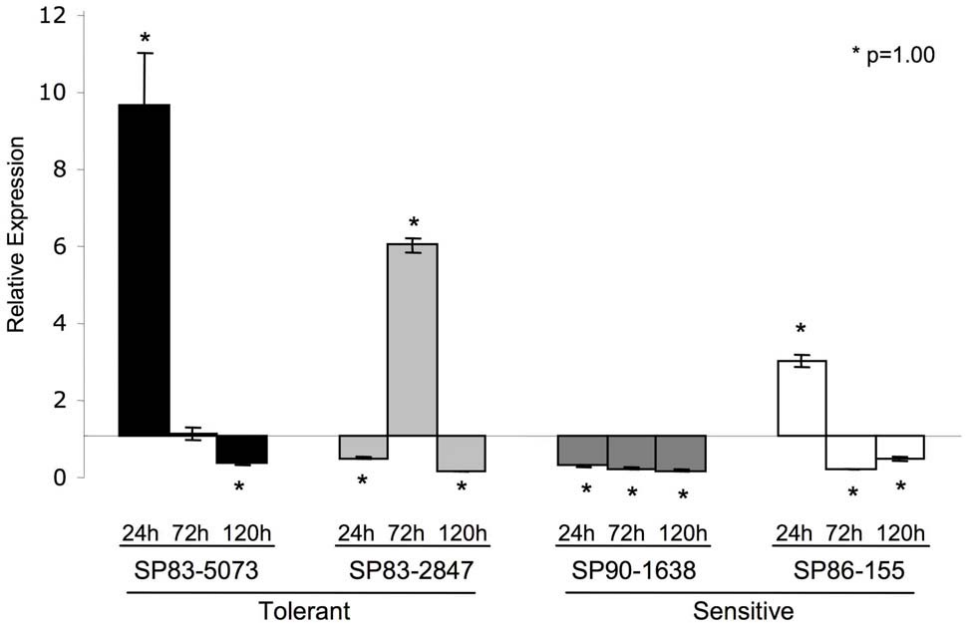
Evaluation of *Scdr1* gene expression in drought-stressed sugarcane plants. *Scdr1* gene expression was evaluated in four sugarcane varieties (SP83-5073, SP90-1638, SP83-2847 and SP86-155) after 24, 72 and 120 hours of control or drought stress conditions. The poly-ubiquitin gene was used as a reference gene for normalization. The *Scdr1* relative expression was normalized to the control condition. Samples with a statistically significant difference in expression level are indicated with asterisks.

SAS (Sugarcane Assembled Sequence) SCSGSB1009D11.g, which encodes a protein of unknown function, was identified as a drought-repressed gene in a previous experiment using the drought-sensitive variety SP90–1638 (Rocha et al., 2007). To further evaluate the expression pattern of *Scdr1,* quantitative real-time PCR was conducted using leaves from two drought-tolerant varieties (SP83-5073 and SP83-2847), another drought-sensitive variety (SP86-155) and SP90-1638. Plants were either exposed to drought or to control conditions for 24, 72 and 120 hours, and S*cdr1* was found to be differentially expressed between control and stressed plants in the four sugarcane varieties (figure 1). Interestingly, the two drought-tolerant varieties, SP83-5073 and SP83-2847, showed a strong induction of *Scdr1*. However, the timing of the response was different in these two varieties, peaking after 24 hours in SP83-5073 (9 times more expressed than control plants) and after 72 hours in SP83-2847 (6 times more expressed than control plants). A repression of *Scdr1* expression was observed in the other two experimental time points in both plants. In one of the drought-sensitive varieties, SP86-155, *Scdr1* was induced after 24 hours of stress but to a much lower level (only four times more expressed in response to drought) compared with the two drought-tolerant varieties and its expression was repressed at later time points. In the other drought-sensitive variety SP90-1638, *Scdr1* was down-regulated at all three time points. Although this expression pattern is complex, the higher induction observed in the tolerant varieties suggested that *Scdr1* may be associated with drought tolerance. This result prompted us to further characterize the *Scdr1* gene.

### ScDR1 Protein Sequence Analysis

**Figure 2:**
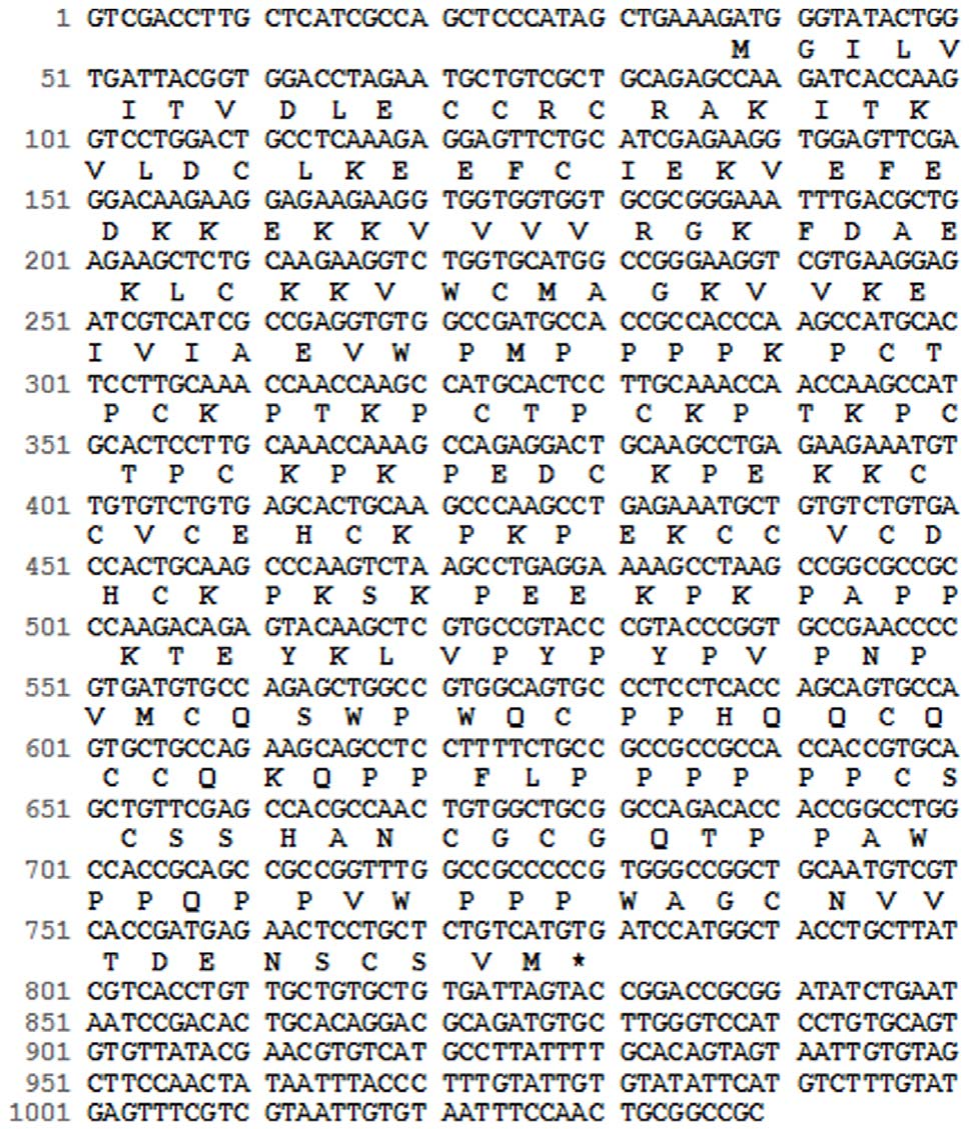
The DNA and deduced protein sequences of *Scdr1 (*Acc. No JN979786). The sequence, corresponding to SAS SCSGSB1009D11.g, was obtained from the SUCEST database.

**Figure 3:**
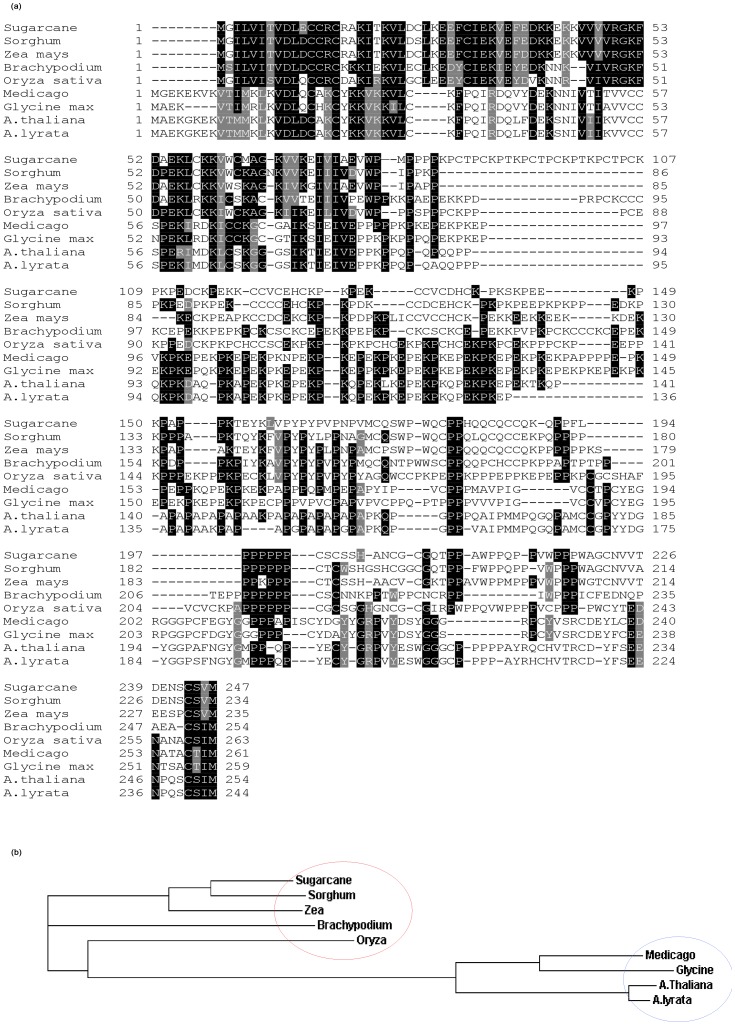
ScDR1 protein sequence analysis. (A) Alignment of ScDR1 with other homolog proteins; (B) Neighbor-joining tree of sugarcane ScDR1 and its homologs in other monocotyledonous and dicotyledonous plants. All sequences were aligned using the Clustal2W software. Bootstrap values are shown as percentages above each node. Sequence accession numbers: sugarcane (Acc. No JN979786), *Sorghum bicolor* (XP_002447741.1), *Zea mays* (ACN37061.1), *Brachypodium distachyon* (XP_003581156), Oryza *sativa* (BAG72124.1), *Medicago trunculata* (ACJ83874.1), *Glycine max* (AAN03471.1), *Arabidopsis thaliana* (NP_974559.5), *A.lyrata (*XP_002870162.1).

The *Scdr1* transcript sequence (744 bp; NCBI Acc. No. JN979786) and deduced protein sequence (248 amino acids) are shown in figure 2. Interestingly, the protein has a high content of proline (19%) and cysteine (13%) residues. To evaluate the similarity of ScDR1 with other plant homologs, an alignment using the complete protein sequence was performed (figure 3a). ScDR1 showed high similarity to homologs from monocotyledonous plants, such as *Sorghum* (89%), maize (84%), rice (70%) and *Brachypodium* (65%). The similarity with proteins from dicotyledonous species was lower and ranged from 29 to 47% in *Arabidopsis thaliana*, soybean and medicago. This alignment was used to construct a neighbor-joining tree (figure 3b). ScDR1 was grouped in the same clade as the analyzed monocotyledonous plants (blue circle), while the dicot plants were grouped in another clade (red circle), consistent with the low sequence similarity (figure 3b).These data indicate that the ScDR1 protein arose prior to the divergence between monocots and dicots.

### Production of Transgenic Plants Overexpressing *Scdr1*

**Figure 4:**
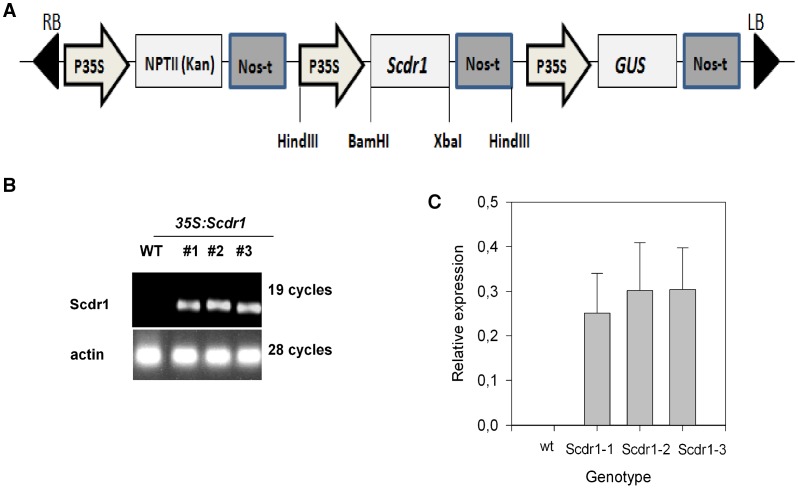
Schematic representation of the pCAMBIA2301::Scdr1 construct and PCR confirmation of plant transgene content. (A) The *Scdr1* coding region was cloned between the constitutive CaMV 35S promoter (P35S) and the NOS polyadenylation signal (Nos-t) using pCambia2301 as the backbone. The *nptII* (kanamycin resistance) gene is also driven by the p35S promoter. LB and RB correspond to the left and right borders of the T-DNA, respectively. The positions of some restriction sites are indicated. (B) Expression of *Scdr1* in WT and three T3-generation transgenic lines. Total RNA was extracted from two-week-old seedlings and then analyzed using semi-quantitative RT-PCR. The actin gene was used as an internal standard. (C) Densitometric analysis of the semi-quantitative RT-PCR.

Transgenic tobacco plants were produced to evaluate the functional role of *Scdr1*. The *Scdr1* coding sequence was PCR amplified and cloned under the control of the cauliflower mosaic virus 35S promoter using pRT104 (Töpfer et al., 1987) as an intermediary vector and pCambia 2301 as the final vector (figure 4a). The construct was expressed in *Agrobacterium tumefaciens* to obtain transgenic tobacco plants. Kanamycin-resistant transformants were further confirmed using an immunohistochemical assay for beta-glucuronidase activity using X-Gluc as a substrate. Seven plants that showed a strong blue staining (data not shown) were selected. Transgenic T0 plants were self-pollinated to obtain homozygous lines. Transformed T1 seedlings were selected using MS medium supplemented with kanamycin (50 mg mL^−1^) and used to obtain homozygous T3 plants. To confirm the integration of the *Scdr1* gene, we performed PCR using genomic DNA as template. *Scdr1-*specific primers were used to amplify a 744 bp fragment using genomic DNA from T3 transgenic plants. The *Scdr1* fragment was detected in transgenic plants; while no amplification was observed in the DNA from wild-type (WT) plants (data not shown). Three independent lines that tested positive for the presence of *Scdr1* by PCR and that showed X-Gluc staining were chosen to evaluate the effects of *Scdr1* overexpression. The expression of *Scdr1*in these lines was confirmed by semi-quantitative RT-PCR (figure 4b, c).

### Effects of *Scdr1* on Seed Germination

**Figure 5:**
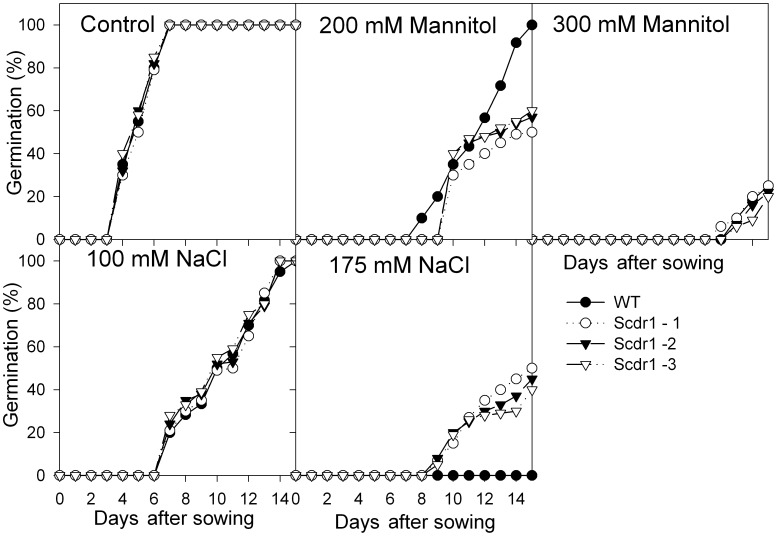
The effects of mannitol and NaCl on seed germination. The percent germination of transgenic (*Scdr1*) and WT tobacco seeds at different concentrations of mannitol or NaCl was evaluated over 15 days.

Seeds from WT and *Scdr1-*transgenic lines were germinated in culture media containing mannitol or NaCl to evaluate the response to drought stress and salt stress, respectively. Under control conditions, similar germination rates were observed between WT and transgenic *Scdr1* plants (figure 5a). In the presence of 200 mM mannitol, *Scdr1* seeds showed reduced germination rates (60%) compared with WT seeds (100%) (figure 5b). Conversely, at higher mannitol concentration (300 mM), the germination of both transgenic and WT seeds was profoundly reduced, reaching only 20% (figure 5c) or completely inhibited (400 mM, data not shown). When seeds were germinated in 100 mM NaCl, no differences were observed between *Scdr1* and WT (figure 5d). However, at a higher salt concentration (175 mM NaCl), while germination was completely inhibited in WT seeds, 50% of the *Scdr1* seeds were capable of germination (figure 5e). These results indicate that *Scdr1* plays a role in protecting against salt stress, but not drought stress, during seed germination.

### Effects of *Scdr1* on Plant Growth

**Figure 6:**
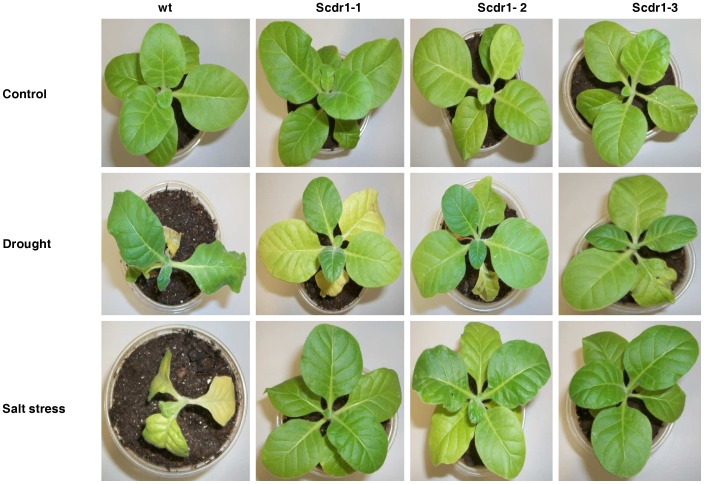
The effects of mannitol and NaCl on tobacco plants. First row: A WT plant and three transformants overexpressing *Scdr1* were grown under control conditions for 13 weeks. Middle row: plants watered with 200 mM mannitol for 10 days and then irrigated with water for 3 days. Bottom row: plants irrigated for 10 days with 175 mM NaCl and then irrigated with water for 3 days.

To analyze the effects of *Scdr1* on plant growth, five-week-old plants were watered with 200 mM mannitol or 175 mM NaCl for 10 days and then allowed to recover for 3 days, during which they were watered with pure water. Under control conditions (watering with pure water for all 13 days), both WT and *Scdr1* plants performed equally well (figure 6). Drought and salt stress caused obvious negative effects in WT plants, which exhibited wilted leaves (figure 6). In contrast, drought and salt tolerance were observed in the *Scdr1*-overexpressing transgenic tobacco plants, indicating that*Scdr1* had a protective role against these two abiotic stresses.

**Figure 7:**
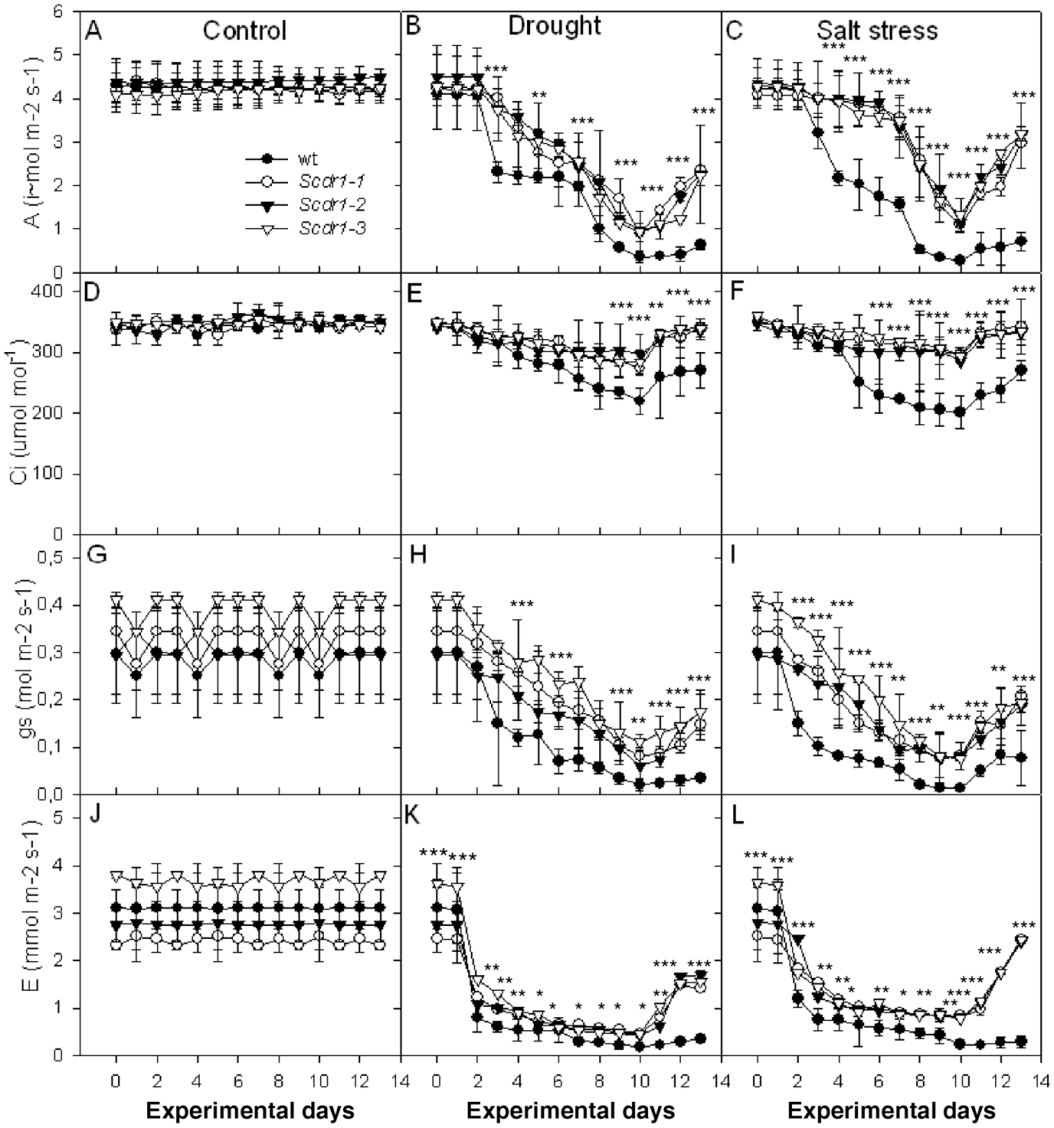
The effects of stress on gas exchange parameters in WT and *Scdr1* transgenic plants. Thirty-day-old plants were exposed for 10 days to 200 mM mannitol or 175 mM NaCl and then allowed to recover for three days by watering with pure water, as described for figure 7. A–C: Net photosynthesis (A); D–F: Internal leaf CO_2_ concentration (Ci); G–I: Stomatal conductance (gs); J–K: Transpiration rate (E). A, D, G and J: Control treatment; B, E, H and K: 200 mM mannitol (drought); C, F, I and L: 175 mM NaCl (salt). Asterisks (***, ** and *) indicate significant differences compared with WT plants in each treatment and each time point (*P*<0.0001, *P*<0.001 and *P*<0.01, respectively, n = 5).

To characterize the performance of the transgenic plants at the physiological level, WT plants and the three independent lines containing the *Scdr1* gene were analyzed using an infrared gas analyzer (IRGA). The obtained transpiration rate (E), net photosynthesis (A), stomatal conductance (gs), internal leaf CO_2_ concentration (Ci) and respiration (R) data are presented in figure 7. Photosynthesis, which is one of the most important parameters involved in plant productivity, was affected in both transgenic and WT plants. In the initial days, no differences between transgenic and WT plants were observed. Beginning on the third day of stress, transgenic plants maintained higher photosynthesis levels than WTs. Following the recovery period, transgenic plants that had been exposed to drought recovered 50% of their initial net photosynthesis (figure 7b), and 75% recovery was observed in previously salt-stressed plants (figure 7c).The WT and s*cdr1* transgenic plants exhibited no significant differences in net photosynthesis under control growth conditions (figure 7a).

The Ci was similar in WT and transgenic plants under control conditions (figure 7d); however, a slight difference of around 20% was observed during drought (figure 7e), and a greater difference of around 50% was observed during salt stress (figure 7f). The gs showed a similar pattern to that of photosynthesis, with *Scdr1* plants less affected by drought and salt stress (figure 7h, i). Similar transpiration rate (E) values were found between the plant lines under normal growth conditions (figure 7j), and both drought and salt stress caused a strong decrease in E values in both lines. *Scdr1* overexpression allowed plants to recover E values after re-watering (figure 7k, l).

**Figure 8:**
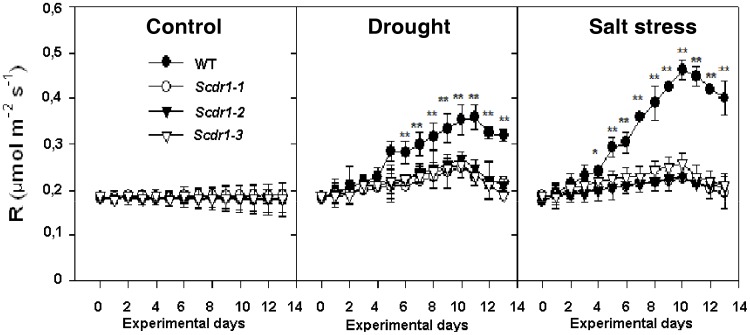
Respiration in tobacco leaves exposed to drought and salt stress. Thirty-day-old WT and transgenic plants were exposed for 10 days to 200 mM mannitol or 175 mM NaCl and were then allowed to recover for three days by watering with pure water. The data shown represent the means of three replicate measurements. Asterisks (** and*) indicate significant differences relative to WT plants in each treatment and each time point (*P*<0.0001 and *P*<0.01, respectively, n = 5).

R in the *Scdr1* and WT plants was similar under control conditions. In both WT and *Scdr1* plants, R increased in response to drought and salt stress, but *Scdr1-*transgenic plants were less affected (figure 8). WT plants showed a two-fold increase in R under salt stress compared with control conditions, while *Scdr1* plants showed only a 25% increase in R. These results suggest that WT plants increase R as a strategy to maintain homeostasis under stress. In contrast, R did not increase in *Scdr1* plants, due to their higher tolerance.

**Figure 9:**
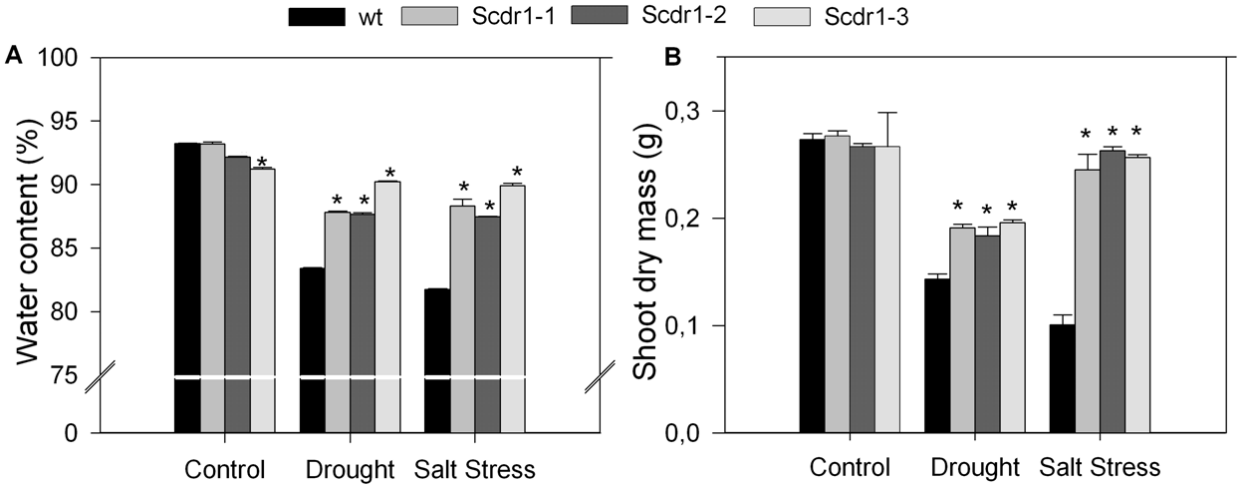
Water content and biomass in WT and *Scdr1* transgenic plants. Thirty-day-old plants were exposed to 200 mM mannitol or 175 mM NaCl for 10 days and then allowed to recover for 3 days by watering with pure water. Control plants were irrigated with water only. The water content in leaves (A) and shoot dry matter (B) were evaluated. Bars represent the means of three independent experiments. Asterisk (*) indicates significant differences compared with WT plants in each treatment (*P*<0.001, n = 5).

Drought and salt stress reduced the water content in both WT and *Scdr1-*transgenic plants. While in the WT plants, water content was reduced from 94% to 83% due to drought stress and to 82% in salt-stressed plants, in *Scdr1* plants these values ranged from 88 to 90%, indicating that a smaller reduction was observed in the transgenic plants (figure 9a). These results indicate that plants overexpressing *Scdr1* were capable of maintaining turgidity under stress, which suggests the occurrence of osmotic adjustment and/or the activation of another mechanism that prevents cellular dehydration. Shoot dry matter was evaluated as an additional parameter to compare WT and *Scdr1* transgenic plants (figure 9b). Under control conditions, WT and *Scdr1* plants had a dry mass of around 0.27 g. Drought and salt stress decreased dry mass in WT plants to 0.15 g and 0.1 g, respectively. *Scdr1* plants were less affected due to drought (dry mass on average of 0.2 g in the three events) and salt-stress (0.26 g). Therefore, although both stresses affected the amount of shoot dry matter in transgenic and WT plants, the reduction was more severe in WT plants than in *Scdr1* plants.

### Analysis of Resistance to Oxidative Stress in *Scdr1* Transgenic Plants

**Figure 10:**
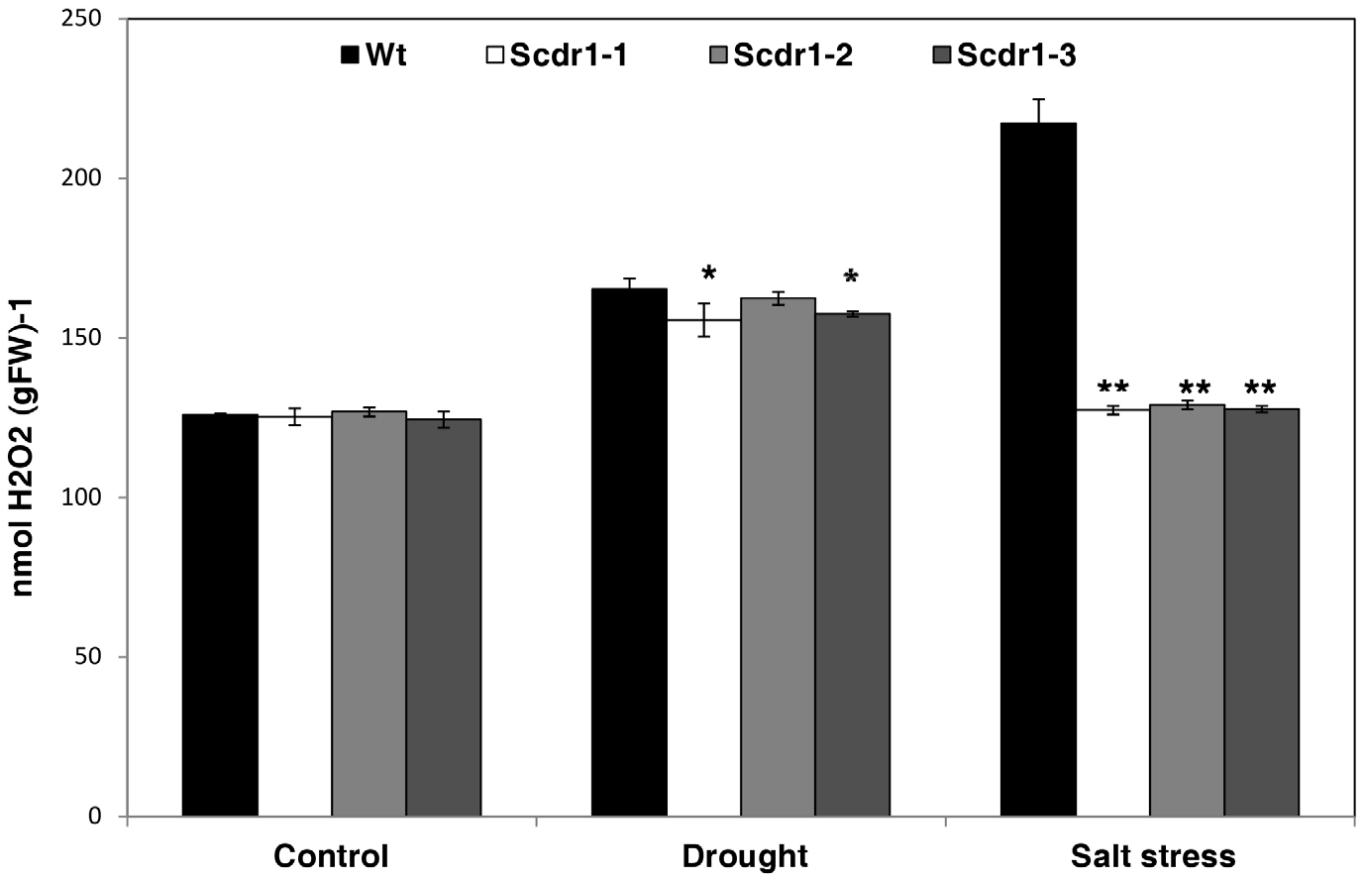
Quantification of hydrogen peroxide in tobacco leaves. Thirty-day-old plants were exposed for 10 days to 200 mM mannitol or 175 mM NaCl. Control plants were irrigated with water. H_2_O_2_ levels in WT and *Scdr1* transgenic lines were determined using Fe-Xylenol orange. Data are represented as the mean±standard deviation from three independent experiments (n = 5). Asterisks (** and *) indicate significant differences compared with WT plants in each treatment (*P*<0.0001 and *P*<0.001, respectively).

To further characterize the role of *Scdr1* on tolerance to abiotic stress, we evaluated the accumulation of H_2_O_2_ in the leaves of transgenic and WT seedlings. Under control conditions, both transgenic and WT plants showed similar H_2_O_2_ levels (figure 10). Following 10 days of drought stress, H_2_O_2_ levels increased similarly in both transgenic and WT plants. Under salt stress, H_2_O_2_ levels increased dramatically in WT plants, reaching 217 nmol H_2_O_2_/gFW, while *Scdr1* transgenic plants were substantially less affected, presenting 127 to 129 nmol H_2_O_2_/gFW in the three independent transgenic events (figure 10). Because H_2_O_2_ levels are correlated with ROS production, we can infer that *Scdr1* plants produced less ROS than WT plants under salt stress.

**Figure 11:**
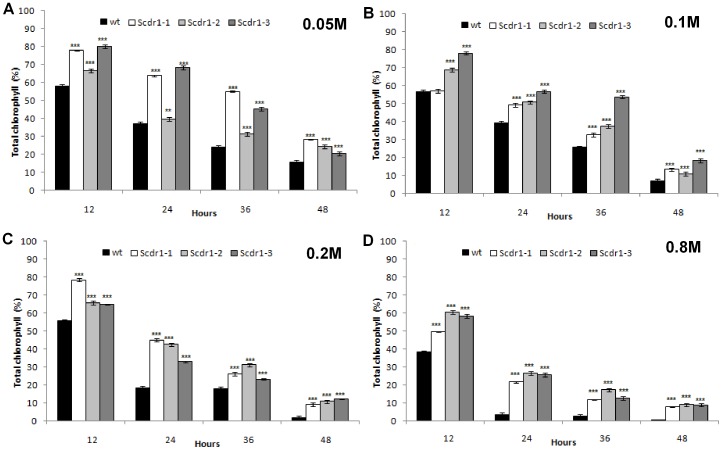
Spectrophotometric quantification of the chlorophyll content in WT and Scdr1 plants exposed to oxidative stress. Leaf discs (1 mm) from three independent *Scdr1* transgenic lines (Scdr1-1, Scdr1-2, Scdr1-3) and non-transgenic control plants were treated with water (control) or with different concentrations of H_2_O_2_: A) 0.05 M, B) 0.1 M_,_ C) 0.2 M and D) 0.8 M. The total chlorophyll content in acetone extracts of H_2_O_2_-treated leaf discs was evaluated spectrophotometrically. Error bars were calculated from three independent experiments (n = 5). Asterisks (*** and **) indicate significant differences compared with WT plants in each treatment (*P*<0.0001 and *P*<0.001 respectively).

To further evaluate the role of *Scdr1* in oxidative stress, leaf discs were exposed to different concentrations of H_2_O_2_ for up to 48 hours (figure 11). Even when the lowest H_2_O_2_ concentration was used (0.05 M), *Scdr1* plants showed a higher percentage of total chlorophyll at all time points (figure 11a). As the H_2_O_2_ concentration was raised to 0.1 and 0.2 M, the differences between WT and *Scdr1* plants increased. The highest concentration (0.8 M) affected WT plants severely, with almost no chlorophyll noted after 48 hours, while *Scdr1* transgenic plants maintained approximately 10% of the initial levels (figure 11d). These results indicate that constitutive overexpression of the *Scdr1* gene in transgenic tobacco plants enhanced tolerance to oxidative stress, allowing the preservation of a higher percentage of chlorophyll.

### Carbon Isotope Discrimination and Photosynthesis in Stressed Plants

**Figure 12:**
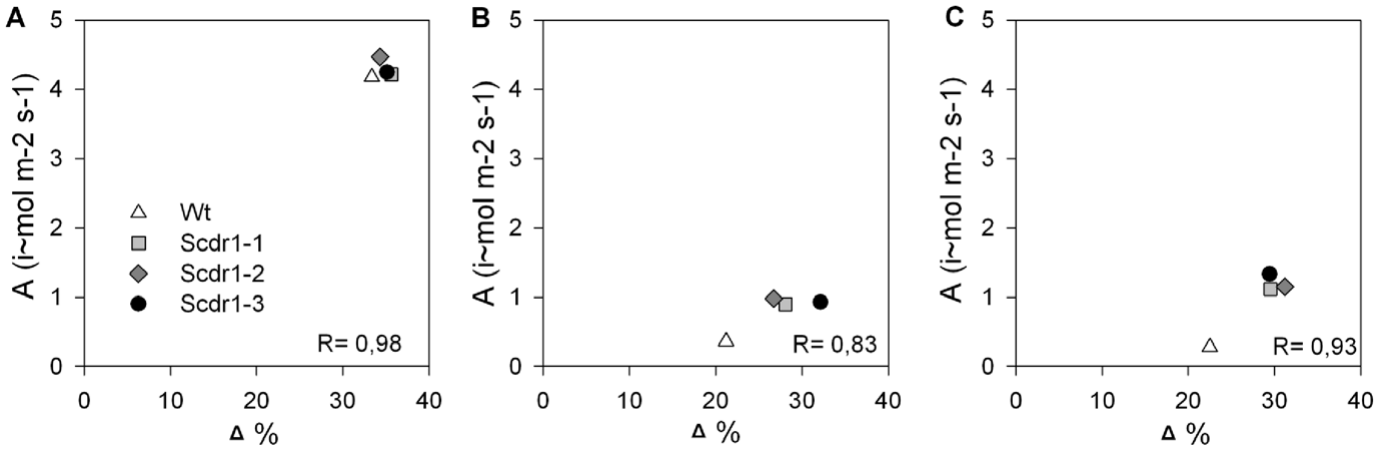
The relationship between net photosynthetic rate *(A)* and carbon isotope discrimination (Δ) in transgenic *Scdr1* and WT plants following 10 days of stress. (A) control, (B) drought, (C) salt stress.

Under environmental stress conditions, there is variation in carbon assimilation that affects photosynthesis (A), mainly due to stomatal limitations. To detect this variation, the relationship between carbon isotopic discrimination (CID) and photosynthesis (*A*) under well-irrigated, drought or salt-stress conditions was investigated. Under well-irrigated conditions, no significant differences were observed between *Scdr1* and WT plants, because both had A rates around 4 i∼mol m^−2^ s^−1^and CID around 34% (figure 12a). CID decreased to 21–22% due to drought and salt stress in WT plants, while in *Scdr1* plants this decrease was smaller (up to 30%). Although *A* had a strong decrease in both plants, WT plants had values around 0.5 i∼mol m^−2^ s^−^1 and Scdr1 plants had A rates in the range from 1–1.3 i∼mol m^−2^ s^−1^ (figure 12b–c). The higher CID and *A* levels in *Scdr1* plants correlated with lower levels of plant stress (figure 6).

## Discussion

Major environmental stresses, such as drought and salinity, contribute to the gap between actual and potential crop yields. To guarantee a sustainable crop yield, it is imperative to design and develop better crop varieties with inbuilt tolerance to the harmful effects of constantly changing environmental factors. Many novel sugarcane stress-induced genes putatively linked to drought and salt stress have been identified [20], [21], but their function in the stress response remains unknown. In this study, a novel drought stress-responsive sugarcane gene (*Scdr1*) was characterized functionally.

Different responses to abiotic stresses are the result of cooperative interactions between multiple physiological, biochemical and morphological features. These interactions may vary between species and even varieties, as observed for drought stress in other plant species [22], [23], [24] and for the *Scdr1* gene in this study in sugarcane (figure 1). The expression of *Scdr1* was different between sensitive and tolerant sugarcane varieties exposed to drought stress. As shown in figure 1, drought-induced *Scdr1* expression was high in two drought-tolerant varieties, down-regulated in one sensitive variety and exhibited only one peak of induction in another sensitive variety. Although we are currently unable to explain the highly complex regulation of this gene, the overexpression of *Scdr1* in transgenic tobacco plants supported the hypothesis that *Scdr1* is involved in sugarcane defense against drought stress. These data raise the question of how gene expression patterns can be used to select genes that are amenable to biotechnological improvement. Certainly, several genes with complex expression patterns, such as *Scdr1*, may have been ignored in functional assays.

The deduced amino acid sequence of the Scdr1 sugarcane protein (figure 1) has similarity to several proteins, all of which are of unknown function (figure 2). The ScDR1 protein has 19% proline and 13% cysteine residues, and no conserved domains were found in this protein. The amino acid sequence of ScDR1 was similar to that of other monocot proteins, and it appeared in a separate clade from that of the dicotyledonous plants (figure 3). This separation suggests an early evolutionary origin for this gene, prior to the divergence of monocots and dicots and followed by an independent evolution inside each clade that might reflect the differences found between the species evaluated. However, the fact that the sugarcane *ScDR1* has conferred drought and salt tolerance to tobacco plants demonstrated that the metabolic pathway in which this gene operates is conserved between monocots and dicots.

In numerous crop plants, the stages of seed germination and early seedling growth are the most susceptible to abiotic stresses [25], [26]. For example, some environmental factors, such low temperature, high concentrations of salt or water deficit, significantly delay the onset of germination and reduce the rate of seed germination events [27], [28], [29], [30]. Transgenic plants overexpressing *Scdr1* showed a greater than 20% germination rate under high salt conditions, while WT seeds did not germinate at all. This result agrees with several previous studies [27], [31], [32], [33] and shows that salt stress is an important limiting factor for germination in different crop species. Here, we showed that transgenic tobacco plants that overexpress *Scdr1* exhibit increased tolerance to salt stress during seed germination and early seedling development (figure 5).

Three independent homozygous *Scdr1* transgenic tobacco lines subjected to multiple stresses, including mannitol (drought), NaCl (salt stress) or H_2_O_2_ (oxidative stress), developed healthy seedlings, in contrast to WT plants, which developed injured and debilitated seedlings (figure 6). Photosynthesis parameters, including rates of transpiration, net photosynthesis, stomatal conductance and internal leaf CO_2_ concentration, were less affected by salt or drought stress in transgenic plants (figure 7). These results demonstrate the unequivocal contribution of *Scdr1* in affording abiotic stress tolerance at the whole-plant level.

The effects of drought stress on plant respiration vary according to the severity of the stress and between species [34], [35]. The percentage of fixed carbon that is respired is predicted to be higher in water-stressed plants because drought typically causes a relatively greater inhibition of photosynthesis than of plant respiration [36]. The increase in respiration observed in WT plants could reflect a plant strategy to increase ATP levels to repair the damage caused by drought and salt stress, as we [37] have shown in drought-stressed tobacco leaves (reviewed by Atkin and Macherel [38]). Because *Scdr1* overexpression reduced the harmful effects of environmental stresses, the need for higher respiration rates was reduced (figure 8).

Environmental stresses affect photosynthetic parameters directly (net photosynthesis) or indirectly (stomatal closure) due primarily to the increased production of oxidative stress-induced ROS [35], [39]. As shown in figure 10, under drought or salt stress, transgenic *Scdr1* tobacco plants showed lower levels of H_2_O_2_ than WT plants. Under optimal conditions, ROS are produced at a low level in organelles with specialized compartments (mitochondria, chloroplasts and peroxisomes) in which a variety of metabolic activities takes place. However, a dramatic increase in ROS levels occurs when cells are submitted to drought, salinity or osmotic stress [35], [40]. Thus, some researchers have suggested that high tolerance to environmental stresses, and drought and salinity in particular, may be associated with a strong defense against oxidative stress at the subcellular and cellular levels [41]. To achieve this tolerance, the whole plant may employ several different strategies [42], [43]. One of these is to control ROS production.

During oxidative stress, *Scdr1* transgenic plants showed higher total chlorophyll content than WT plants (figure 11). All pathways that activate mechanisms leading to ROS scavenging have been shown to play an important role in protecting plants against different abiotic stresses [35], [44]. Because the overexpression of *Scdr1* in transgenic tobacco plants enhanced tolerance to drought, salt and oxidative stress, a putative role for the ScDR1 protein is the avoidance of ROS production under harmful conditions. The elucidation of the exact pathway leading to this protection mechanism requires further study.

During photosynthesis, plants discriminate against ^13^C. Under stressful conditions, ^13^C discrimination is affected by stomatal limitations and the enzymatic processes of photosynthesis [45]. Different studies [46], [47], [48] have demonstrated that carbon isotope discrimination (CID) is highly correlated with plant water-use efficiency. Measurements of CID in C3 and C4 plants have also been used as integrated measures of the photosynthetic gas exchange response to environmental variables, such as drought [49], [50], [51], [52] and salinity [53], [54], [55]. As shown in figure 12, WT plants showed less CID and A during drought and salt stress, which correlated with lower stomatal conductance and consequently reduced absorption of CO_2_, as reviewed by Chaves et al. [39].

It is worth to note that although *Scdr1* overexpression had a positive impact in several plant parameters either under drought or salt stress, major impacts were observed under salt stress. Interestingly, in WT plants the increase in the H_2_O_2_ levels was much higher due to salt stress compared to drought stress, and *ScDR1* overexpression decreased oxidative stress at a higher level in salt-stressed plants compared to the drought stressed ones (Figure 10). Therefore, we believe that this action on oxidative stress could explain the better effect of ScDR1 in protecting plants from salt stress.

In summary, our data shed light on the role of a novel sugarcane gene in drought and salt stress response. *Scdr1* encodes a protein of unknown function, and our data suggest that *Scdr1* is involved in protecting cells and the whole plant against the stress-induced accumulation of ROS. Our study highlights the relevance of the group of genes that encode unknown proteins, which make up a large portion of most genomes. *Scdr1* has the potential to be used in biotechnological applications to produce sugarcane varieties with greater tolerance to both drought and salt stress. Future work will focus on understanding the pathways controlled by this gene.

## Materials and Methods

### Plant Material and Growth Conditions

Seeds of wild-type (WT) tobacco (*Nicotiana tabacum, var. SR1*) were germinated in Petri dishes containing Murashige-Skoog (MS) medium with 0.9% (w/v) agar. Seedlings were transplanted to soil consisting of Bacto (Michigan Peat Co., Houston) and sand (4∶1, v/v) in a 325 mL pot. Plants with 10 to 12 leaves were transplanted to 1-L containers of the same mixture and maintained in a growth chamber with a 16/8 h light/dark cycle (300–400 µmol photons m^−2 ^s^−1^) at 25°C and a relative humidity of 75–80%. Under these conditions, WT tobacco remains in the rosette stage until at least 5 weeks of age.

Sugarcane plants from two drought-tolerant (SP83-5073 and SP83-2847) and two drought-sensitive varieties (SP90-1638 and SP86-155) were grown in greenhouses as described by Rocha et al. [20]. Briefly, plants were grown in pots containing moist sand and watered with Hoagland’s solution [56] for 5 weeks. Water was then withheld, and leaves were collected after 24, 72 and 120 h. Control plants were irrigated normally. Leaf samples were stored at −80°C.

### Quantitative Real-time PCR

Quantitative real-time PCR conducted as described by Rocha et al. [20]. Total RNA was isolated from leaf samples taken from drought-stressed and non-stressed plants. Poly-ubiquitin was used as the reference gene [20]. Relative expression (experimental/control) was determined using the 2^−ΔΔC^_t_ method [57], and a sample not subjected to stress was used as a control. For the statistical analysis of relative gene expression, we assumed a log-normal model that calculates the probability Pr (sample> reference) and Pr (sample <reference) for up- and down-regulated genes, respectively. The expression profile was considered validated when P≥0.95. Leaves from six plants were used for each time point per treatment.

### Construction of Plant Expression Vectors

The complete coding sequence of *Scdr1* (Acc. No JN979786) was cloned from SAS SCSGSB1009D11.g, which was obtained from the Brazilian Clone Collection Center (BCCCENTER, Brazil). The template DNA was amplified using PCR with gene-specific primers (Forward: 5′-GGATCCCTCATCGCCAGCTCCCAT-3′ and reverse: 5′-TCTAGACCTGTGCAGTGTCGGATTATTC-3′), cloned into pGEMT-Easy (Promega, USA) and then introduced between the BamHI and XbaI sites of the pRT104 vector [58]. The resulting expression cassette, under the control of the 35S promoter and using the NOS terminator, was transferred as a HindIII fragment into the pCAMBIA 2301 vector (Cambia, Australia). The resulting construct (pCAMBIA2301::*Scdr1*) was introduced into *Agrobacterium tumefaciens* strain GV3101 (Clontech, USA).

### Semi-quantitative RT-PCR

Total RNA was extracted using the RNeasy Plant Mini Kit (Qiagen, USA), and first-strand cDNA was synthesized from 2 µg of total RNA using the Superscript III Kit (Invitrogen, USA) with oligo d(T)18 primers according to the manufacturer’s instructions. Semi-quantitative RT-PCR was performed with 1 µl of cDNA in a 25 µl reaction volume. The PCR conditions for the amplification of *Scdr1* were as follows: 1 min at 94°C, followed by 30 cycles of 45 s at 94°C, 60 s at 53°C and 75 s at 72°C. The same conditions were used for the amplification of the WT tobacco actin gene, except that the number of PCR cycles were decreased to 28 and the Tm used was 60°C. The PCR products were examined using electrophoresis on a 1% agarose gel. The experiment was repeated three times, and relative densitometric ratios were determined using ImageJ (http://rsbweb.nih.gov/ij/).

### Transformation of Tobacco Plants

Leaves from WT tobacco plants were surface-sterilized, cut into small discs and incubated with *A. tumefaciens* suspensions for 5–10 min. Plant tissues were then transferred to MS medium (supplied with 2 mg L^−1^ benzyladenine and 0.1 mg L^−1^ naphthylacetic acid) for 3 days. Selection was conducted using a selection medium (MS salts, 2 mg L^−1^ benzyladenine, 0.1 mg L^−1^ naphthylacetic acid, and either 100 mg L^−1^ kanamycin and 500 mg L^−1^ carbenicillin), and developing shoots were then transferred to MS medium containing 0.1 mg L^−1^ indole-3-acetic acid and either 100 mg L^−1^ kanamycin and 500 mg L^−1^carbenicillin. Plants with roots were then transferred to soil and maintained in a growth chamber as described above.

### Seed Germination Assays

Seeds from transgenic and WT tobacco plants were surface-sterilized with 70% (v/v) ethanol for 1 min, incubated in a 2% (v/v) NaClO solution for 30 min and rinsed 5–6 times in sterile distilled water. Seeds were sown in Petri dishes (30 seedlings/dish) containing MS medium and incubated in a chamber at 23°C with a 16/8 h light/dark cycle (300–400 µmol photons m^−2^ s^−1^). Different concentrations of mannitol (0, 200, 300 and 400 mM) or NaCl (0, 100 and 175 mM) were used to induce drought and salt stress, respectively. The number of germinated seeds was measured daily.

### Drought and Salt Stress Assays in Tobacco Plants

Seeds from WT *Nicotiana tabacum* and three independent and homozygous T3-generation transgenic lines (Scdr1-1, Scdr1-2 and Scdr1-3) were allowed to germinate for 16 days and were then grown in pots filled with Plantmax HT (Eucatex, Brazil). The pots were placed in a growth chamber at 22°C with an 18-hour light period/day. Plants were irrigated with 70 ml of water daily for 4 weeks prior to stress treatments. For drought or salt stress, seedlings were irrigated with 70 ml of 200 mM mannitol or 175 mM NaCl, respectively, for 10 days and were then allowed to recover with pure water irrigation for 3 days, as described by Zhang et al. [59]. Five plants were used for each treatment.

We used completely expanded leaves at the same positions on the tobacco plants to estimate the stomatal conductance of CO_2_ (gs), transpiration rate (E) and net photosynthetic rate (A). Measurements were taken with an Infrared Gas Analyzer (IRGA, LCpro+; ADC Bioscientific, UK) at a CO_2_ concentration of 360 µL L^−1^, a saturating light intensity of 1000 µmol m^−2 ^s^−1^ and a gas flow rate of 200 mL min^−1^. The temperature inside the leaf chamber was 25°C [60].

To perform respiration (R) measurements, 5-week-old plants were grown in a chamber at 25°C with a 16/8 h light/dark photoperiod. To avoid artifacts caused by transient metabolic activities following darkening, known as light-enhanced dark respiration, measurements of night respiration were performed after 3 hours of acclimation to darkness. Carbon dioxide production was measured with an IRGA, as described by Begcy et al. [37].

### Hydrogen Peroxide Determination

A modified ferrous ammonium sulphate/xylenol orange (FOX) method was used to quantify H_2_O_2_ [61]. Briefly, 300 mg of leaves (fresh material) from WT and *Scdr1* transgenic plants that were well irrigated and treated with mannitol or salt for 10 days, as described previously, was subjected to methanol extraction (1∶5 w/v mg sample/mL) at 0°C. The lead samples were ground in a mortar and then centrifuged at 10,000×*g* for 5 min. We then mixed 100 µL of supernatant, 500 µL of 1 mM Fe(NH_4_)_2_(SO_4_)_2_ and 200 µL of 250 mM HSO_4_. The reaction mixture was incubated in the dark for 5 min. Xylenol orange (100 µL, 1 mM) was then added, and the mixture was again incubated in the dark for 20 min. H_2_O_2_ donates electrons to Fe, which in turn binds to xylenol to form a purple compound. A standard curve of known concentrations of H_2_O_2_ (0, 2.5, 5, 7.5, 10, 12.5 and 15 µM H_2_O_2_) was generated and used to quantify the contents of the sample.

### Carbon Isotope Discrimination

We used leaves from the same plants that were used for H_2_O_2_ quantification. The carbon isotope composition of dry leaf samples was determined using ratio mass spectrometry in the Laboratório de Isótopos Estáveis, Centro de Energia Nuclear na Agricultura (CENA), Universidade de São Paulo (Piracicaba, Brazil),as described by Chandra and Bhatt [62]. Δ13C was calculated according to the protocol of Williams et al. [51] from plant Δ^13^C values measured under drought, salinity or control conditions (*n = *3).

### H2O2 Treatment and Total Chlorophyll Determination

Oxidative stress was induced as described by Brandalise et al. [63]. Fully-expanded leaves from WT and three independent transgenic *Scdr1* plants were grown for 5 weeks in a growth chamber at 23°C with a 16/8 h light/dark cycle (300–400 µmol photons m^−2^ s^−1^). Leaf discs (1 cm in diameter) were cut and floated on 0, 0.05, 0.1, 0.2, 0.4 or 0.8 M H_2_O_2_ for 12, 24, 36 or 48 h at 25°C under constant light in three independent experiments. The degree of oxidative stress in treated leaf tissues was determined spectrophotometrically as the total chlorophyll content in leaf discs following extraction in acetone at 4°C, as described by Arnon [64].

### Statistical Analysis

For statistical calculations, the mean values, standard deviation and *t*-test values were computed using pre-loaded software in Excel (http://www.Physics.csbsju.edu/stats/t-test.html).
